# Fluorescence-excitation and Emission Spectroscopy on Single FMO Complexes

**DOI:** 10.1038/srep31875

**Published:** 2016-08-22

**Authors:** Alexander Löhner, Khuram Ashraf, Richard J. Cogdell, Jürgen Köhler

**Affiliations:** 1 Experimental Physics IV and Bayreuth Institute for Macromolecular Research (BIMF), University of Bayreuth, Germany; 2 Institute of Molecular, Cell & Systems Biology, College of Medical Veterinary and Life Sciences, University of Glasgow, United Kingdom

## Abstract

In green-sulfur bacteria sunlight is absorbed by antenna structures termed chlorosomes, and transferred to the RC via the Fenna-Matthews-Olson (FMO) complex. FMO consists of three monomers arranged in C_3_ symmetry where each monomer accommodates eight Bacteriochlorophyll *a* (BChl *a*) molecules. It was the first pigment-protein complex for which the structure has been determined with high resolution and since then this complex has been the subject of numerous studies both experimentally and theoretically. Here we report about fluorescence-excitation spectroscopy as well as emission spectroscopy from individual FMO complexes at low temperatures. The individual FMO complexes are subjected to very fast spectral fluctuations smearing out any possible different information from the ensemble data that were recorded under the same experimental conditions. In other words, on the time scales that are experimentally accessible by single-molecule techniques, the FMO complex exhibits ergodic behaviour.

Photosynthesis is one of the most important energy conversion processes on Earth. The generic principle of photosynthesis is that sunlight is absorbed by antenna complexes and then this energy is transferred efficiently to the photochemical reaction centre that acts as a transducer. Typically, both the antennae and the reaction centre (RC) are pigment-protein complexes accommodating a number of pigments that are properly positioned with respect to each other by the interaction with a protein scaffold. For many of these complexes the structural arrangement has been elucidated with high resolution^1^,^2^,^3^,^4^,^5^,^6^,^7^,^8^,^9^, stimulating enormous interest in the investigation of the structure-function relationships of these systems^10^. Next to ultra-fast time-resolved^11^,^12^,^13^,^14^,^15^ and high-resolution spectroscopy^16^,^17^,^18^,^19^,^20^,^21^,^22^,^23^ single-molecule techniques in particular have contributed tremendously to our current understanding of the electronic structure of those pigment-protein complexes^24^,^25^,^26^,^27^,^28^,^29^,^30^.

In green-sulfur bacteria sunlight is absorbed by antenna structures termed chlorosomes (a unique case where no proteins are involved) and transferred to the RC via the Fenna-Matthews-Olson (FMO) complex, which is thought to play an important role in unidirectional energy transfer^31^. As a matter of fact the FMO complexes of the bacterial species *Chlorobium limicola* and *Prosthecochloris aestuarii* were the first photosynthetic antenna complexes for which a high-resolution x-ray structure was determined^32^,^33^. Meanwhile the structure of the FMO complex from the species *Chlorobaculum (Cba.) tepidum* also became available^34^, and resembles closely the former one. Accordingly, this FMO complex consists of three identical subunits that are arranged in C_3_ symmetry with respect to each other, such that the C_3_ axis is perpendicular to the membrane axis, Fig. 1a. Each subunit contains 7 closely interacting bacteriochlorophyll (BChl) *a* molecules and an additional 8^th^ BChl *a* pigment that is located in a region directed towards the chlorosome^35^, Fig. 1b,c. Numerous studies including linear spectroscopy, linear and circular dichroism, hole burning, transient absorption, and photon echo spectroscopy flanked by theoretical work and computer simulations have been performed on this system (reviewed in ref. 31), making this complex one of the most widely studied pigment-protein complexes so far. Unfortunately, the site energies of the pigments cannot be deduced directly from the ensemble absorption spectrum which features rather broad bands. As a consequence of this, the available information about the excitonic coupling between the BChl *a* pigments is based on theoretical calculations and/or numerical fits of the optical spectra, and the outcome is sensitive to the choice of the input parameters. Although the models discussed differ slightly in their details there is agreement that the electronic coupling between the seven BChl *a* in close proximity leads to the formation of exciton states that are delocalised over only a few monomers^36^. Upon excitation the decay from the highest to the lowest exciton state occurs on a (sub-)picosecond time scale, and the population can be transferred either by a few big steps or by many small steps involving all intermediate exciton states. Finally, the excitation energy leaves the FMO complex en route to the RC via BChl *a* number “3” (“exit pigment”), see Fig. 1b,c.

Recently, by applying 2D spectroscopy, long-lived quantum coherences among the electronic excitations of the pigments in the FMO complex have been observed^37^,^38^, and it has been argued that these reflect correlations of the site energy fluctuations. A technique that might shed some light on this debate is single-molecule spectroscopy, because, in general, the broadening of the spectra due to averaging over sample heterogeneities can be avoided. Indeed, there is a demand for experiments on single FMO complexes in the community and challenging experimental schemes have been proposed recently^39^. Here we present the fluorescence-excitation and emission spectra from individual FMO complexes from the species *Cba. tepidum* and find that these complexes behave ergodically on the experimental accessible timescales.

## Results

**Figure 2:**
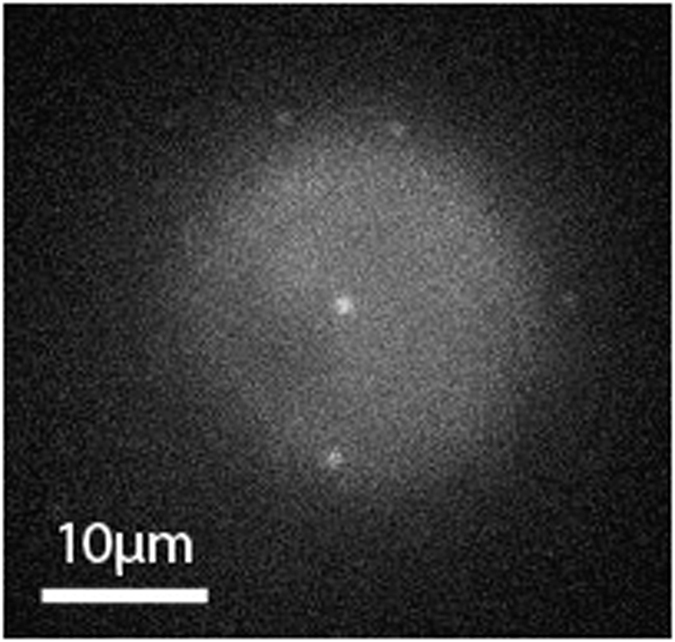
Widefield image of a sample with an FMO concentration of 320 pM. For excitation the output of the laser was wobbled between 800 nm and 809 nm at a rate of 0.3 s^−1^. The excitation intensity was 175 W/cm^2^, and the acquisition time was 15 s.

In order to select a single well-separated FMO for fluorescence-excitation spectroscopy we recorded a widefield fluorescence image from the sample. An example for such an image is shown in Fig. 2. It features a few diffraction-limited bright spots, each corresponding to an individual FMO complex.

Evidence that we are dealing with a single FMO complex stems from the following observations: First, for 320 pM concentration and a diffraction-limited excitation volume for the laser spot this yields a probability of 0.15 for finding a single FMO within this spot. Given that the FMO complexes have no tendency to aggregate, the probability to find two FMOs within the same volume is already less than 0.02. Based on these numbers one expects an average distance between two FMO complexes of about 15 μm, which is consistent with the widefield images of the samples. Second, for a single complex the emission rate n corresponding to the number of emitted photons per second is given by 
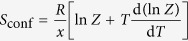
. Here σ denotes the absorption cross section, A the area of the focal spot, P the incident laser power, hν the photon energy and Φ_F_ the fluorescence quantum yield. Inserting, σ = 5.88 × 10^−20^ m^2^ (calculated from the extinction coefficient = 154 mM^−1^cm^−1^ 40), A = 6.36 × 10^−13^ m^2^, P = 80 nW, hν = 2.48 × 10^−19^ J, and Φ_F_ = 0.95, we expect a fluorescence emission rate of about 29,000 photons/s. From previous work we know that the collection efficiency of our low-temperature microscope is about 2%, which yields 600 counts/s for the detected rate from a single FMO, in agreement with our observations.

**Figure 3:**
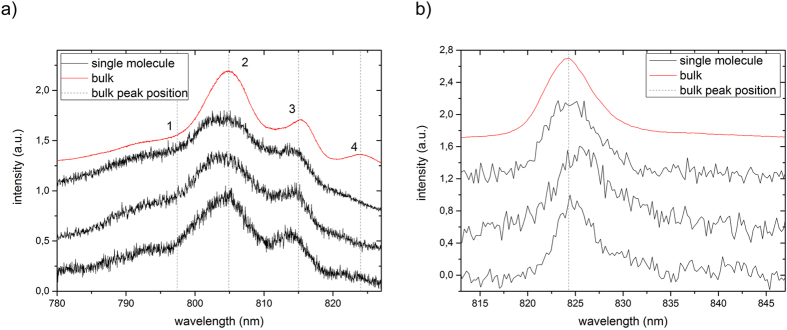
Low-temperature spectra (1.2 K) of the FMO complex from *Chlorobaculum tepidum.* (**a**) Fluorescence-excitation spectra of FMO from an ensemble (red) and three different single complexes (black). All spectra were recorded with an excitation intensity of 12.5 W/cm^2^. The dashed vertical lines indicate the peak positions of the four Gaussians that have been used for fitting the bulk spectrum. For better comparison the spectra have been normalised, and are offset with respect to each other. The typical signal strength at the maximum amounts to 400 to 600 counts per second. Each spectrum corresponds to an average of 200 individual spectra recorded with an acquisition time of 19 s each. (**b**) Fluorescence emission spectra from an ensemble (red) and three different individual FMO complexes (black). The excitation wavelength was 805 nm, and the excitation intensity was 250 W/cm^2^. For better comparison the spectra have been normalised, and are offset with respect to each other. Each spectrum corresponds to an average of 400 individual spectra recorded with an acquisition time of 5 s each.

Figure 3 compares the low-temperature static optical spectra of FMO from *Cba. tepidum* for a bulk sample and three different single complexes. In accordance with absorption spectra in the literature, the fluorescence-excitation spectrum from an ensemble (Fig. 3a, red line) features four bands, here labelled 1–4 in the order of increasing wavelength. Those can be characterised by four Gaussians (for details see experimental section) that decrease in widths going from band 1 to band 4, see Table 1. The corresponding spectrum from the single FMO complexes (Fig. 3a, black lines) in shape resemble closely the ensemble spectrum, with the exception that band 4 shows a lower relative intensity with respect to the ensemble spectrum and is sometimes hard to detect. In order to verify whether the single complex spectrum is affected by the excitation intensity we repeated the experiment for one single complex lowering and raising the excitation intensity by a factor of 5 and 4 respectively. The lower excitation intensity corresponds to the lowest intensity for which we can record an excitation spectrum. For these different excitation intensities we did not find a significant variation of the spectral profile.

Figure 3b displays the low-temperature emission spectra from three other individual FMO complexes together with the emission spectrum from a bulk sample. The samples have been excited at 805 nm and the excitation intensity was 250 W/cm^2^. Again we find a close resemblance between the ensemble and the single complex spectra. The emission spectrum features a single band that can be characterised by a Gaussian shape with a width of about 80 cm^−1^ (FWHM). For comparison, with one FMO complex we also recorded an emission spectrum with a reduced excitation intensity of 50 W/cm^2^. This resulted in an extremely noisy emission spectrum without significant change of its width. Details concerning the emission spectra are summarised in Table 2.

## Discussion

The general finding is that there is no observable significant difference in the peak positions found in the linear optical spectra recorded for an ensemble or individual FMO complexes. We do, however, find a slight variation in the widths of these bands between individual complexes. Heating effects by the laser as observed for example in ref. 41 in the past as an origin of the line broadening can be excluded. In their work the samples were excited with pulses of 2 ps duration at 1 mJ/cm^2^ corresponding to a peak excitation intensity of 500 MW/cm^2^. The concomitant rise in temperature was then estimated to be about 10 K if the energy is distributed over a group of seven BChl *a* molecules and 500 K if the energy is concentrated, for example by annihilation processes, on a single BChl *a* molecule. Here the highest excitation intensity used is 250 W/cm^2^, i.e. more than 6 orders of magnitude lower than in the previous study. The widths of these bands, as predicted by theoretical work^42^, are determined mainly by the relaxation of the exciton states and/or fast fluctuations of the site energies of the individual pigments. Superimposed on this major contribution to the line-widths is a small additional broadening due to the heterogeneities (static and/or dynamic) between the complexes. Relaxation dynamics as the dominant origin of the line-widths is in line with the observation that the widths of the absorption bands decrease upon going from high- to low energy in the spectral positions of these bands. This is because the higher energy exciton states have more relaxation pathways available, which therefore accelerates their decay with respect to the low energy exciton states. The observed bandwidths of 70–400 cm^−1^ provide a lower limit for the relaxation times of 10 and 70 fs, which is in agreement with previous work^43^,^44^. Similarly, previous theoretical work predicts fluctuations of the site energies of the individual pigments on the same time scale^42^,^45^,^46^. Obtaining a spectrum from a single FMO complex requires that the signal is collected for a finite dwell time, here 10 ms, in order to achieve a sufficient signal-to-noise ratio. Hence, a spectrum recorded from an individual FMO complex corresponds to a time-averaged spectrum, where the period over which the signal has been accumulated is very long with respect to the (predicted) time scale of the energetic fluctuations. In other words: A single FMO complex behaves ergodically on the experimentally accessible time scale. However, this single molecule study does reveal an important basic property of the internal energy transfer relaxation processes that take place within FMO, and it clarifies what kind of experiments with single FMO complexes can be done at all and what the relevant timescales are.

## Methods

### Sample preparation

FMO was prepared from cells of the thermophilic green sulphur bacterium *Cba. tepidum.* A strain containing a His-tagged version of the green sulphur bacterium *Cba. tepidum* reaction centre (RC) was a gift from the group of Prof. Oh-Oka^47^. *Cba. tepidum* was grown anaerobically in the light in modified Pfennig’s medium^48^, known as liquid CL media^47^,^49^. Glycerol stocks of the *Cba. tepidum* cells were inoculated in CL media and grown in 1.2 L air tight bottles that were allowed to go anaerobic by leaving them in the dark for 12 hours overnight. Cells were then grown at 43 °C under illumination for 2–3 days, at a light intensity of approximately 30 μmol photons m^−2^ s^−1^, before harvesting by centrifugation (12,000 x g). The RC was purified as described in ref. 50 with the exception of the cell breakage, which was carried out by using a cell disrupter (25 psi). At the stage of nickel affinity chromatography a high salt wash with >500 mM NaCl elutes FMO. This dilute solution of FMO was then purified by ion exchange chromatography on Whatman DE52 cellulose. FMO eluted between 90–200 mM NaCl. It was then concentrated by ultrafiltration with a 50 kDa MWCO membrane (Minicon concentrator, Millipore) and further purified, in 20 mM Tris HCl, pH8, by size exclusion chromatography on a Sepharose S-200 (GE Healthcare) column^51^.

The FMO complexes were stored in a 20 mM Tris A buffer (pH 8.0 at room temperature) at −80 °C until they were used. For single-molecule measurements the samples were diluted within three steps in a 20 mM Tris A buffer to a concentration of 320 pM. In the last dilution step the pre-diluted sample was mixed with the same amount of pure glycerol to avoid crystallisation at low temperatures. A small drop (0.5 μl) of this solution was seeped onto a cleaned SiO_2_ plate before the drop was covered with a microscope coverslip to get a flat sample surface. The prepared sample was put immediately into the cryostat, pre-filled with liquid nitrogen to flash freeze the sample before it was cooled down to 1.2 K. For bulk measurements the sample preparation was identical, except for the FMO concentration which was 800 μM.

### Fluorescence-Excitation Spectroscopy

For the fluorescence-excitation experiments the samples are excited with the output from a titan-sapphire-laser (3900 S, Spectra Physics) that was pumped by a Nd:YVO_4_ laser (Millenia Vs, Spectra Physics). The wavelength is varied between 770 nm and 827 nm by turning the birefringent filter with a stepper motor (Actuator 850 F, Motion Controller MM4005, Newport). The accuracy and the reproducibility of the wavelength variation is 1 cm^−1^, as verified with a wavemeter (WaveMaster, Coherent). The excitation light passes a home-build fluorescence microscope and is focussed by an objective (Mikrothek, NA = 0.85) that is mounted inside the cryostat to a diffraction-limited spot of 0.9 μm in diameter. For the selection of an individual complex the microscope is operated in wide-field mode and the laser wavelength is wobbled between 800 nm and 809 nm. The emission from the sample is collected by the same objective, transmitted through two band-pass filters (LP830, AHF Analysetechnik) for suppressing residual laser light, and detected with a CCD-camera (iKon, Andor). From the widefield image (Fig. 2) a spatially well separated FMO complex is selected and the microscope is switched to confocal mode. A similar setup is described in great detail in refs 52,53. The signal passes the bandpass filters mentioned above and is registered with a single-photon counting avalanche photodiode (APD) (SPCM-AQR-16, Perkin Elmer). During acquisition of the excitation spectra, the intensity of the laser is recorded as a function of the wavelength using a powermeter (LaserMate-Q, Coherent), and all spectra are corrected for variations of the laser intensity.

Since in the literature the FMO spectra are mostly given on a wavelength scale we have chosen to display the spectra in Fig. 3 on a wavelength scale. However, for fitting the data we used an energy equivalent axis (wavenumbers) on the abscissa and took the required intensity correction into account for the conversion of the spectral peak positions from wavenumbers to the corresponding wavelengths.

### Emission Spectroscopy

For emission spectroscopy the sample is excited at 805 nm, and the light from the sample is guided through a spectrometer (Acton 250, Princeton Instruments) that accomodates a mirror and a grating (300 lines/mm, blaze 1000 nm). For selecting a single FMO complex the setup is operated in wide-field mode and the emission from the sample is directed onto a CCD -camera (iKon, Andor) via the mirror in the spectrometer. From the widefield image a single FMO complex is selected, the microscope is switched to confocal mode as before, and the mirror in the spectrometer is replaced by the grating. For registering the spectra with the CCD, 16 pixel were binned vertically to reduce the read-out noise. For a single complex 400 spectra were recorded successively with a dwell time of 5 s, which yields a total acquisition time of 30 minutes. Since the individual spectra did not feature any fluctuations on these time scales we display the sum spectrum in Fig. 3b.

## Additional Information

**How to cite this article**: Löhner, A. *et al*. Fluorescence-excitation and Emission Spectroscopy on Single FMO Complexes. *Sci. Rep.* **6**, 31875; doi: 10.1038/srep31875 (2016).

## Figures and Tables

**Figure 1:**
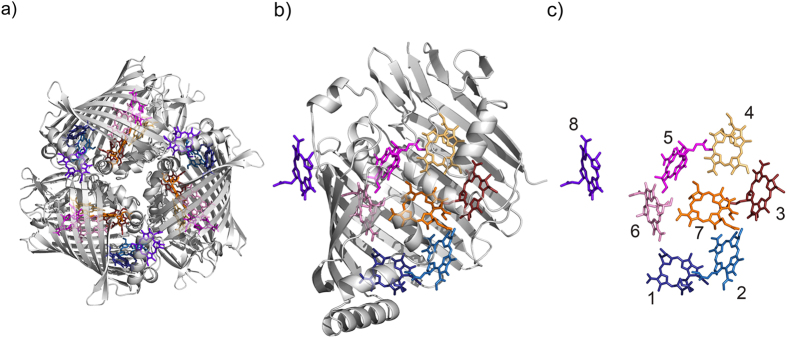
Structure of the FMO complex. (**a**) Top-view of the trimeric FMO complex from *Chlorobaculum tepidum*. (**b**) Side view of the BChl *a* arrangement within one monomer unit. For clarity the phytol tails have been omitted. (**c**) Same as (**b**) without the protein scaffold. The delocalisation of the excitation over two pigments is indicated by similar colour tones (violet, blue, orange). Structural data taken from www.rcsb.org (protein code 3ENI).

**Table 1:** Spectral positions and bandwidths observed in the fluorescence-excitation spectra displayed in Fig. 3a.

		Bulk	complex 1	complex 2	complex 3
Peak 1	position (nm)	797.4 ± 0.5	795 ± 2	794 ± 2	796 ± 2
	FWHM (cm^−1^)	355 ± 8	490 ± 15	260 ± 15	316 ± 15
Peak 2	position (nm)	804.9 ± 0.5	804.8 ± 0.5	804.6 ± 0.5	804.5 ± 0.5
	FWHM (cm^−1^)	99 ± 2	160 ± 5	152 ± 5	133 ± 5
Peak 3	position (nm)	814.9 ± 0.5	814.5 ± 0.5	814.2 ± 0.5	814.0 ± 0.5
	FWHM (cm^−1^)	71 ± 2	88 ± 5	100 ± 5	75 ± 5
Peak 4	position (nm)	824.0 ± 0.5			
	FWHM (cm^−1^)	52 ± 2			

The spectral peaks have been numbered 1–4 in the order of increasing wavelength. For the single-complex spectra a meaningful analysis of the positions and widths of peak 4 is prevented by the low signal-to-noise ratio.

**Table 2:** Spectral positions and bandwidths observed in the emission spectra displayed in Fig. 3b.

	Bulk	complex 4	complex 5	complex 6
position (nm)	824.4 ± 0.6	824.6 ± 0.8	826.0 ± 0.8	825.1 ± 0.8
FWHM (cm^−1^)	97.0 ± 1.5	99 ± 7	114 ± 7	72 ± 7

## References

[b1] Deisenhofer J., Epp O., Miki K., Huber R. & Michel H. Structure of the protein subunits in the photosynthetic reaction centre of *Rhodopseudomonas viridis* at 3 Angsgtröm resolution. Nature 318, 618 (1985). 22439175 10.1038/318618a0

[b2] Ermler U., Fritzsch G., Buchanan S. K. & Michel H. Structure of the photosynthetic reaction centre from *Rhodobacter sphaeroides* at 2.65 Å resolution: cofactors and protein-cofactor interactions: Structure. Structure 2, 925–936 (1994). 7866744 10.1016/s0969-2126(94)00094-8

[b3] Papiz M. Z., Prince S. M., Howard T. D., Cogdell R. J. & Isaacs N. W. The Structure and Thermal Motion of the B800-850 LH2 Complex from *Rps. acidophila* at 2.0 (A)Over-Circle Resolution and 100 K: New Structural Features and Functionally Relevant Motions. J. Mol. Biol. 326, 1523–1538 (2003). 12595263 10.1016/s0022-2836(03)00024-x

[b4] McDermott G. . Crystal structure of an integral membrane light-harvesting complex from photosynthetic bacteria. Nature 374, 517–521 (1995).

[b5] Ferreira K. N., Iverson T. M., Maghlaoui K., Barber J. & Iwata S. Architecture of the Photosynthetic Oxygen-Evolving Center. Science 303, 1831–1838 (2004). 14764885 10.1126/science.1093087

[b6] Loll B., Kern J., Saenger W., Zouni A. & Biesiadka J. Towards complete cofactor arrangement in the 3.0 Å resolution structure of photosystem II. Nature 438, 1040–1044 (2005). 16355230 10.1038/nature04224

[b7] Zouni A. . Crystal Structure of Photosystem II From *Synechococcus Elongatus* at 3.8 Ångstrom Resolution. Nature 409, 739–743 (2001). 11217865 10.1038/35055589

[b8] Jordan P. . Three-Dimensional Structure of Cyanobacterial Photosystem I at 2.5 Angstrom Resolution. Nature 411, 909–917 (2001). 11418848 10.1038/35082000

[b9] Amunts A., Drory O. & Nelson N. The structure of a plant photosystem I supercomplex at 3.4 Angström resolution. Nature 447, 58–63 (2007). 17476261 10.1038/nature05687

[b10] Sauer K. . Structure based calculations of the optical spectra of the LH2 bacteriochlorophyll-protein complex from *Rhodopseudomonas acidophila*. Photochem. Photobiol. 64, 564–576 (1996).

[b11] Zigmantas D. . Two-Dimensional Electronic Spectroscopy of the B800-B820 Light-Harvesting Complex. PNAS 103, 12672–12677 (2006). 16912117 10.1073/pnas.0602961103 PMC1568908

[b12] Collini E. . Coherently wired light-harvesting in photosynthetic marine algae at ambient temperature. Nature 463, 644–647 (2010). 20130647 10.1038/nature08811

[b13] Harel E. & Engel G. S. Quantum coherence spectroscopy reveals complex dynamics in bacterial light-harvesting complex 2 (LH2). PNAS 109, 706–711 (2012). 22215585 10.1073/pnas.1110312109 PMC3271880

[b14] Fidler A. F., Singh V. P., Long P. D., Dahlberg P. D. & Engel G. S. Time Scales of Coherent Dynamics in the Light-Harvesting Complex 2 (LH2) of *Rhodobacter sphaeroides*. JPC Let. 4, 1404–1409 (2013). 10.1021/jz400438m PMC3714110 23878622

[b15] Brixner T. . Two-diemsional spectroscopy of electronic couplings in photosynthesis. Nature 434, 625–628 (2005). 15800619 10.1038/nature03429

[b16] Purchase R. & Völker S. Spectral hole burning: examples from photosynthesis. Photosynth. Res. 101, 245–266 (2009). 19714478 10.1007/s11120-009-9484-5 PMC2744831

[b17] Freiberg A., Rätsep M., Timpmann K., Trinkunas G. & Woodbury N. W. Self-Trapped Excitons in LH2 Antenna Complexes between 5 K and Ambient Temperature. JPC B 107, 11510–11519 (2003).

[b18] Rätsep M. & Freiberg A. Resonant Emission From the B870 Exciton State and Electron-Phonon Coupling in the LH2 Antenna Chromoprotein. CPL 377, 371–376 (2003).

[b19] Reddy N. R. S., Small G. J., Seibert M. & Picorel R. Energy transfer dynamics of the B800-B850 antenna complex of *Rhodobacter sphaeroides*: a hole burning study. CPL 181, 391–399 (1991).

[b20] Wu H. M., Reddy N. R. S. & Small G. J. Direct observation and hole burning of the lowest exciton level (B870) of the LH2 antenna complex of *Rhodopseudomonas acidophila* (strain 10050). JPC B 101, 651–656 (1997).

[b21] Wu H. M., Rätsep M., Jankowiak R., Cogdell R. J. & Small G. J. Hole burning and absorption studies of the LH1 antenna complex of purple bacteria: Effects of pressure and temperature. JPC B 102, 4023–4034 (1998).

[b22] Caro C. de., Visschers R. W., van Grondelle R. & Völker S. Spectral hole burning in pigment protein complexes of photosynthetic bacteria. J. Lumin. 58, 149–153 (1994).

[b23] Creemers T. M., Caro C. de., Visschers R. W., van Grondelle R. & Völker S. Spectral hole burning and fluorescence line narrowing in subunits of the light harvesting complex LH1 of purple bacteria. JPC B 103, 9770–9776 (1999).

[b24] Cogdell R. J., Gall A. & Köhler J. The architecture and function of purple bacteria: from single molecules to *in vivo* membranes. Q. Rev. Biophys. 39, 227–324 (2006). 17038210 10.1017/S0033583506004434

[b25] van Oijen A. M., Ketelaars M., Köhler J., Aartsma T. J. & Schmidt J. Unraveling the electronic structure of individual photosynthetic pigment-protein complexes. Science 285, 400–402 (1999). 10411501 10.1126/science.285.5426.400

[b26] Jelezko F., Tietz C., Gerken U., Wrachtrup J. & Bittl R. Single molecule spectroscopy on photosystem I pigment protein complexes. JPC B 104, 8093–8096 (2000).

[b27] Richter M. F. . Refinement of the x-ray structure of the RC LH1 core complex from *Rhodopseudomonas palustris* by single-molecule spectroscopy. PNAS 104, 20280–20284 (2007). 18077352 10.1073/pnas.0704599105 PMC2154422

[b28] Brecht M., Hussels M., Schlodder E. & Karapetyan N. V. Red antenna states of Photosystem I trimers from *Arthrospira platensis* revealed by single-molecule spectroscopy. BBA-Bioenergetics 1817, 445–452 (2012). 22155210 10.1016/j.bbabio.2011.11.012

[b29] Krüger T. P. J., Wientjes E., Croce R. & van Grondelle R. Conformational switching explains the intrinsic multifunctionality of plant light-harvesting complexes. PNAS 108, 13516–13521 (2011). 21808044 10.1073/pnas.1105411108 PMC3158202

[b30] Hildner R., Brinks D., Nieder J. B., Cogdell R. J. & van Hulst N. F. Quantum Coherent Energy Transfer over Varying Pathways in Single Light-Harvesting Complexes. Science 340, 1448–1451 (2013). 23788794 10.1126/science.1235820

[b31] Milder M., Brüggemann B., van Grondelle R. & Herek J. Revisiting the optical properties of the FMO protein. Photosynth. Res. 104, 257–274 (2010). 20229036 10.1007/s11120-010-9540-1 PMC2882565

[b32] Fenna R. E. & Matthews B. W. Chlorophyll arrangement in a bacteriochlorophyll protein from *Chlorobium limicola*. Nature 258, 573–577 (1975).

[b33] Matthews B. W., Fenna R. E., Bolognesi M. C., Schmid M. F. & Olson J. M. Structure of a bacteriochlorophyll a-protein from the green photosynthetic bacterium *Prosthecochloris aestuarii*. J. Mol. Biol. 131, 259–285 (1979). 490647 10.1016/0022-2836(79)90076-7

[b34] Li Y.-F., Zhou W., Blankenship R. E. & Allen J. P. Crystal structure of the bacteriochlorophyll a protein from *Chlorobium tepidum*. J. Mol. Biol. 271, 456–471 (1997). 9268671 10.1006/jmbi.1997.1189

[b35] Ben-Shem A., Frolow F. & Nelson N. Evolution of photosystem I – from symmetry through pseudosymmetry to asymmetry. FEBS Letters 564, 274–280 (2004). 15111109 10.1016/S0014-5793(04)00360-6

[b36] Louwe R. J. W., Vrieze J., Hoff A. J. & Aartsma T. J. Toward an Integral Interpretation of the Optical Steady-State Spectra of the FMO-Complex of *Prosthecochloris aestuarii*. 2. Exciton Simulations. JPC B 101, 11280–11287 (1997).

[b37] Engel G. S. . Evidence for Wavelike Energy Transfer Through Quantum Coherence in Photosynthetic Systems. Nature 446, 782–786 (2007). 17429397 10.1038/nature05678

[b38] Cheng Y.-C. & Fleming G. R. Dynamics of Light Harvesting in Photosynthesis. Annu. Rev. Phys. Chem. 60, 241–262 (2009). 18999996 10.1146/annurev.physchem.040808.090259

[b39] Tao M.-J., Ai Q., Deng F.-G. & Cheng Y.-C. Proposal for probing energy transfer pathway by single-molecule pump-dump experiment. Scientific Reports 6, 27535 (2016). 27277702 10.1038/srep27535 PMC4899753

[b40] Blankenship R. E., Olson J. M. & Miller M. In Anoxygenic Photosynthetic Bacteria, edited by Blankenship R. E., Madigan M. T. & Bauer C. E. (Kluwer Academic Publishers, Dordrecht, 1995), pp. 399–435.

[b41] Gulbinas V. . Singlet–Singlet Annihilation and Local Heating in FMO Complexes. J. Phys. Chem. 100, 17950–17956 (1996).

[b42] Olbrich C. . From atomistic modeling to excitation transfer and two-dimensional spectra of the FMO light-harvesting complex. JPC B 115, 8609–8621 (2011). 10.1021/jp202619a PMC3140161 21635010

[b43] Savikhin S., Zhou W., Blankenship R. E. & Struve W. S. Femtosecond energy transfer and spectral equilibration in bacteriochlorophyll a–protein antenna trimers from the green bacterium *Chlorobium tepidum*. Biophys. J. 66, 110–114 (1994). 8130329 10.1016/S0006-3495(94)80769-6 PMC1275669

[b44] Savikhin S. & Struve W. S. Ultrafast Energy Transfer in FMO Trimers from the Green Bacterium *Chlorobium tepidum*. Biochemistry 33, 11200–11208 (1994). 7727371 10.1021/bi00203a016

[b45] Wang X., Ritschel G., Wüster S. & Eisfeld A. Open quantum system parameters from molecular dynamics. *arXiv* (2015). 10.1039/c5cp03891j 26372495

[b46] Cho M., Vaswani H. M., Brixner T., Stenger J. & Fleming G. R. Exciton Analysis in 2D Electronic Spectroscopy. JPC B 109, 10542–10556 (2005). 10.1021/jp050788d 16852278

[b47] Azai C. . A heterogeneous tag-attachment to the homodimeric type 1 photosynthetic reaction center core protein in the green sulfur bacterium *Chlorobaculum tepidum*. BBA 1807, 803–812 (2011). 21420930 10.1016/j.bbabio.2011.03.007

[b48] Wahlund T. M., Woese C. R., Castenholz R. W. & Madigan M. T. A thermophilic green sulfur bacterium from New Zealand hot springs, *Chlorobium tepidum* sp. nov. Arch. Microbiol. 156, 81–90 (1991).

[b49] Azai C., Tsukatani Y., Harada J. & Oh-oka H. Sulfur oxidation in mutants of the photosynthetic green sulfur bacterium *Chlorobium tepidum* devoid of cytochrome c-554 and SoxB. Photosynth. Res. 100, 57–65 (2009). 19421892 10.1007/s11120-009-9426-2

[b50] Oh-oka H., Kamei S., Matsubara H., Iwaki M. & Itoh S. Two molecules of cytochrome c function as the electron donors to P840 in the reaction center complex isolated from a green sulfur bacterium, *Chlorobium tepidum*. FEBS Letters 365, 30–34 (1995). 7774710 10.1016/0014-5793(95)00433-a

[b51] Ashraf K. U. D. University of Glasgow (2014).

[b52] Lang E., Baier J. & Köhler J. Epifluorescence, confocal and total internal reflection microscopy for single-molecule experiments: a quantitative comparison. J. Microsc. 222, 118–123 (2006). 16774520 10.1111/j.1365-2818.2006.01579.x

[b53] Hofmann C., Aartsma T. J., Michel H. & Köhler J. Spectral dynamics in the B800 band of LH2 from *Rhodospirillum molischianum*: A single-molecule study. New J. Phys. 6, 1–15 (2004).

